# Carbon Monoxide in Meat and Fish Packaging: Advantages and Limits

**DOI:** 10.3390/foods7020012

**Published:** 2018-01-23

**Authors:** Djamel Djenane, Pedro Roncalés

**Affiliations:** 1 Laboratory of Food Quality and Food Safety, Department of Food Science and Technology, University Mouloud Mammeri, P.O. Box 17, Tizi-Ouzou 15000, Algeria; 2 Laboratory of Meat and Fish Technology, Department of Animal Production and Food Science, Faculty of Veterinary Sciences, University of Zaragoza, C/Miguel Servet, 177, 50013 Zaragoza, Spain; roncales@unizar.es

**Keywords:** muscle foods, modified atmosphere packaging, CO, shelf-life, best practice, regulation

## Abstract

Due to increased demands for greater expectation in relation to quality, convenience, safety and extended shelf-life, combined with growing demand from retailers for cost-effective extensions of fresh muscle foods’ shelf-life, the food packaging industry quickly developed to meet these expectations. During the last few decades, modified atmosphere packaging (MAP) of foods has been a promising area of research, but much remains to be known regarding the use of unconventional gases such carbon monoxide (CO). The use of CO for meat and seafood packaging is not allowed in most countries due to the potential toxic effect, and its use is controversial in some countries. The commercial application of CO in food packaging was not then considered feasible because of possible environmental hazards for workers. CO has previously been reported to mask muscle foods’ spoilage, and this was the primary concern raised for the prohibition, as this may mislead consumers. This review was undertaken to present the most comprehensive and current overview of the widely-available, scattered information about the use of CO in the preservation of muscle foods. The advantages of CO and its industrial limits are presented and discussed. The most recent literature on the consumer safety issues related to the use of CO and consumer acceptance of CO especially in meat packaging systems were also discussed. Recommendations and future prospects were addressed for food industries, consumers and regulators on what would be a “best practice” in the use of CO in food packaging. All this promotes high ethical standards in commercial communications by means of effective regulation, for the benefit of consumers and businesses in the world, and this implies that industrialized countries and members of their regulatory agencies must develop a coherent and robust systems of regulation and control that can respond effectively to new challenges.

## 1. Introduction

Oxidative browning is the primary basis for consumer rejection of fresh beef in retail display. The meat industry has made a great effort to develop techniques that can improve color stability. Consumers prefer that raw meat of good quality is bright red, which is an indicator of freshness [1].

Traditionally in developed countries, fresh meat is wrapped in O_2_-permeable film allowing the meat to turn bright red. This bright red color is retained under these conditions for about a few days (~3 days). The shelf-life of perishable fresh meat is limited in the presence of normal air. Chilled storage will significantly reduce the rate at which detrimental changes occur in the food, but will not extend the shelf-life sufficiently for retail distribution and display purposes.

Over the past decade, the use of case-ready modified atmosphere packaging (MAP) has increased by the meat industry in various countries. Carbon dioxide (CO_2_), nitrogen (N_2_) and oxygen (O_2_) are the gases most commonly used in MAP fresh meats. Indeed, the majority of red meat products are packaged in a high O_2_ environment (~80% O_2_) to reduce myoglobin (Mb) oxidation and provide a stable, attractive, “bloomed” red meat color, in a proportion of at least 20% CO_2_ to prevent the growth of Gram-negative bacteria responsible for aerobic spoilage such as *Pseudomonas* spp. However, high O_2_-MAP (HiO_2_-MAP) can increase lipid and protein oxidation, with negative effects on meat flavor [2,3] and texture, which reduces the tenderness and juiciness of the meat [4,5]. Another concern of HiO_2_ packaging is the possible development of premature browning (PB); the phenomenon develops when meat is cooked, resulting in meat that appears done before it has reached a temperature that renders it microbiologically safe [6,7,8,9], thus causing the risk of consumption of undercooked meat with pathogenic bacteria. 

To extend red color stability and avoid the drawbacks of aerobic packaging, an anaerobic MAP technology with 0.4% CO (CO-MAP) was approved [10] for use with fresh meats in the USA [11]. Previous studies have proven that CO can significantly increase the color stability of beef compared with other packaging methods. The possible reason that CO-MAP could enhance red color stability was related to the higher stability of carboxymyoglobin (COMb) than oxymyoglobin (O_2_Mb) [12,13], owing to the stronger binding of CO to the iron-porphyrin site on the Mb molecule [14]. The main advantages of CO include the maintenance of the desirable attributes mentioned above in relation to color stability, growth reduction of spoilage organisms and prevention of oxidative processes [11]. However, due to the potential toxic effect of CO, its use is controversial in some countries. 

Vacuum packaging (VP) is another common method used to distribute meats and in supermarkets for their retail/display. Storage of beef in gas-impermeable packages confers to product a purple color owing to the formation of deoxymyoglobin (DeoxMb). The anaerobic environment delays aerobic microbial growth such as *Pseudomonas* spp. and psychrotrophic aerobic bacteria and improves microbial shelf-life. However, consumers prefer the appearance of bright red beef compared to the darker vacuum-packaged beef products [15]. It is possible that a pre-treatment with CO will make it possible to overcome the unattractive color in the vacuum-packed meat pieces. This will maintain an attractive red color throughout the exposure and sales period, allowing for a more tender meat due to optimum ripening during this period.

Since 1985, Norwegian meat industries have used 0.4% CO in MAP of muscle foods (fresh beef, pork and lamb) with 60–70% CO_2_ and the balance as N_2_. About 60% of the fresh meat in Norway has been sold using this gas composition [16]. The use of CO for MAP of meat has been prohibited in Norway since 1 July 2004, due to the implementation of European Union (EU) food regulations.

The scope and purpose of this paper and the controversy about the use of CO in meat packaging were discussed. The use of CO in fresh meat packaging gives promising results due to its positive effects on overall meat quality, enhancement red color, reduced lipid oxidation and microorganism growth inhibitions, which result in shelf-life prolongation during wider distribution of case-ready products. However, in realistic concentrations, CO as such has no antimicrobial effect, and CO_2_ in sufficient concentrations is required for delaying the growth of Gram-negative bacteria.

The use of CO in the food industry is controversial. Some countries approve the application such as the U.S., Canada, Australia and New Zealand, while the EU member states ban it from food processing. CO has previously been reported to mask meat spoilage, and this was the primary concern raised for the prohibition as this may mislead consumers. Another consideration is that the application of CO in meat packaging was not considered feasible because of possible environmental hazards for workers. 

Risk of CO toxicity from the packaging process or from consumption of CO-treated meats is negligible. Moreover, the addition of CO pre-treatments prior to VP may be beneficial to allow a desirable color to be induced while allowing aging to occur within the package and increasing meat tenderness. Additionally, CO is not present in the pack during storage. Several authors do not consider CO only as a toxic gas for our organism. It was quickly discovered that CO is produced endogenously as a cellular protectant by nearly every cell in our bodies when they are subjected to situations of oxidative stress or injury. Although there is increasing interest in the use of CO-MAP for fresh beef, the debates during the last few years concerning the use of CO in meat packaging have not seriously taken into account the preferences of consumers [17]. However, several studies on the attitude of European consumers regarding the use of CO for meat packaging have reported positive relationships that suggest its future potential within the EU. Facilitation of information can help to develop future policies to ensure consumer protection, and therefore, the debate over the use of CO as a protective gas in meat packaging within the EU could be re-considered. This review provides not only the CO-MAP technology as the solution for the shelf-life issues of muscle foods, but also the new controversy in the use of CO in meat and fish packaging, with the key arguments of both parties. The impact of this review to the field of academic research, food industries and public health was also discussed.

## 2. Fresh Meat Packaging Methods

### 2.1. Raw Meat Spoilage-Associated Storage Conditions

Fresh meat suffers during refrigerated storage some modifications, which can be either physical (water loss) or chemical (color and odor modification) or microbiological. The shelf-life of fresh meat is not unlimited. As is known, its alteration is due to a greater or lesser extent to the presence of atmospheric O_2_, as a consequence of a series of well-known mechanisms: 1. the oxidizing chemical effect of the atmospheric O_2_; 2. the growth of aerobic spoilage microorganisms; 3. photo-oxidation. All of these factors, either alone or in combination, can result in detrimental changes in the color, odor, texture and flavor of meat. Maintaining meat’s quality attributes throughout its shelf-life has been a perennial challenge for the meat industry. In this context, the best packaging methods in combination with low temperatures have been considered an important technology with respect to maintaining quality standards with optimal distribution and extending shelf-life for the retailers.

#### 2.1.1. Microbial Spoilage

Fresh meat is an excellent source of nutrients and makes it an ideal environment for the growth of spoilage microorganisms and common pathogens. Therefore, it is essential that adequate preservation techniques are applied to maintain its quality and safety. The presence of aerobic conditions results in the growth of mainly aerobic psychrotrophic bacteria types, *Pseudomonas*, *Acinetobacter* and *Moraxella*, responsible for fresh meat spoilage during aerobic cold storage. It has been reported that microbial spoilage of meat occurs when counts of aerobic bacteria reach levels of 7 log_10_ cfu/g [18]. This level is commonly found to be correlated with sensory deterioration, like off-odors and the presence of slime on the surface of meat.

#### 2.1.2. Lipid Oxidation

Lipids are an important component of meat and contribute to its several desirable characteristics. Meat oxidation not only influences the eating quality of the products, but also has harmful effects on the health of humans by the formation of carcinogenic substances. Malondialdehyde (MDA), which is a degradation product of lipid oxidation, has been criticized as a carcinogenic factor in food. The development of rancidity in meat by lipid oxidation begins at the time of slaughter and is strongly enhanced during processing and storage during which the phospholipids are released from the membrane so they can be more easily oxidized. These processing steps disrupt muscle structures, causing unsaturated fatty acids to react with atmospheric O_2_ and to increase contact with endogens macromolecules that promote auto-oxidation in the meat systems. Lipid oxidation might not be considered a limiting factor for beef shelf-life, as it occurs at a slower rate than pigment oxidation or microbial spoilage.

Free radical chain reaction is the mechanism of lipid peroxidation and reactive O_2_ species (ROS) such as hydroxyl radical and hydroperoxyl radical and iron are the major initiators of the chain reaction in the development of lipid peroxidation in meat and meat products.

The formation of volatile lipid oxidation products strongly reduces the consumer’s acceptability of the product in a number of ways, including off-odors and off-flavor formations. Oxidative processes can also affect the ability of the membranes to hold water and may contribute to drip loss and consequently cause changes in the functional and sensory characteristics of the meat [19]. Therefore, the development of lipid oxidation varies due to the livestock species and environmental conditions.

There are, thus, many factors that influence lipid oxidation and off-flavor in muscle foods. The oxidation rate will depends strongly on the presence of O_2_, the pro-oxidants/antioxidants balance, the degree of unsaturated fatty acids and the storage conditions. Control of these factors is the best way to retard lipid oxidation and off-flavor in meat products. Antioxidants and chelating agents are the most effective inhibitors of lipid oxidation [20,21].

#### 2.1.3. Pigment Oxidation

**Figure 1:**
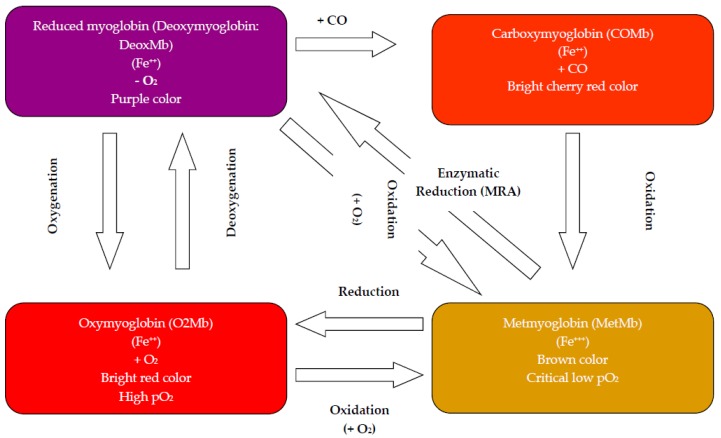
The cycle of color in fresh red meat.

Due to the rejection of fresh discolored beef in retail/display, the meat industry could suffer financial losses estimated at several millions of dollars and according to Mancini and Hunt [13], and a 15% price reduction of retail meat is reported due to surface discoloration. The redness of meat color is produced by oxygenation of DeoxMb to O_2_Mb, due to exposure to O_2_. This oxygenation is considered reversible according to the partial pressure of O_2_ (pO_2_). Discoloration of the meat surface results from oxidation of pigment to metmyoglobin (MetMb). Often, the oxidation of pigment is only slowly reversible by enzyme-mediated reduction of MetMb in the early stages of pigment oxidation (Figure 1). Therefore, preservation of meat color appearance involves primarily the prevention or slowing of MetMb formation on exposed meat surfaces.

The heme group of Mb contains an iron atom within a protoporphyrin ring. The color that Mb imparts to meat is determined by the redox status of its heme iron and the chemical species bound to heme. DeoxMb is most commonly observed in fresh beef slices just after cutting from whole muscle. The relative concentration of O_2_ to which Mb is exposed is critical to the redox form it will assume. When O_2_ is present at approximately 4–6 mmHg, the MetMb form will predominate [22]. This is a very important relationship and must be considered in light of natural O_2_-consumption that may occur from mitochondrial activity, lipid oxidation and/or bacterial growth. Overall, studies demonstrated that O_2_ consumption concomitant with *P*. *fluorescens* growth decreased pO_2_, which accelerated O_2_Mb oxidation and consequently beef discoloration. Meat appearance in retail/display is influenced by several factors such as species and muscle location, marbling, aging period, display lighting and temperature [23]. Hydrogen sulfide (H_2_S) is presumably produced by contaminating bacteria, binding heme and forming the green pigment, sulfmyoglobin (SHb). Lipid oxidation is positively correlated with pigment oxidation [18,24,25,26]. The correlation of lipid oxidation with a decrease in redness is related to the formation of precursors for O_2_Mb oxidation from lipid oxidation, and ferric hemes are believed to promote lipid oxidation. Generally, consumers discriminate beef containing 20% MetMb on the surface [27], and it is rejected from purchase when MetMb levels exceed 40% [28]. Microbial contamination could play a role in the phenomenon of meat surface discoloration by competition for O_2_ and possibly reduce pO_2_ below the critical level for oxidation of pigment to MetMb [29]. 

#### 2.1.4. Photooxidation

In retail cases, displaying meat under lights accelerates the formation of MetMb, which produces unattractive brown color. The effect of light on lipid and pigment oxidation has been demonstrated in meat systems [28]. UV-light (UV-A) is more effective than visible light in inducing oxidation of lipid and pigments [30,31].

Discoloration of fresh beef is significantly faster upon light exposure than when kept in the dark, an observation of particular importance for retail display [28]. The dull appearance associated with light-induced Mb decay has been recognized for decades [32], but only a few mechanistic details have been elucidated. The fluorescent tubes usually used in supermarkets for meats’ retail/display have emission spectra designed to maintain a color balance. In a study on the effect of different sources of light on packaged meat quality, Djenane et al. [28] reported that the level of MetMb on the surface of the meat displayed under the standard tube (Mazdafluor Aviva TF/36 w, Philips, Eindhoven, The Netherlands) can reach 70% of MetMb on the 17th day of display, while that exposed to the Promolux® tube (Platinum L36 w, Market Group Ventures Inc., Shawnigan Lake, BC, Canada) or in the presence of a UV filter (polycarbonate) can reach only 40% of MetMb for 28 days of display. Standard fluorescent tubes used in display cabinets may transmit radiation below 400 (UV-A ~390 nm), so it must be taken into account for its deleterious effects on meat display life [33]. Nevertheless, light discoloration depends on many technological factors (temperature, pO_2_, meat pH, storage time, free transition metal ions, light wavelengths, type of display lighting, atmosphere) that can affect the photocatalyzed autoxidation [34]. The effect of light on lipid oxidation has been demonstrated in various food systems [35,36,37,38]. Lee et al. [39] found that lipid oxidation was influenced by light intensity (1000 vs. 3000 lux). Herbs and spices that are usually added to sausages and processed meats may contain chlorophyll. The latter can absorb light and promote lipids’ photo-oxidation.

### 2.2. Fresh Meat Shelf-Life

**Figure 2:**
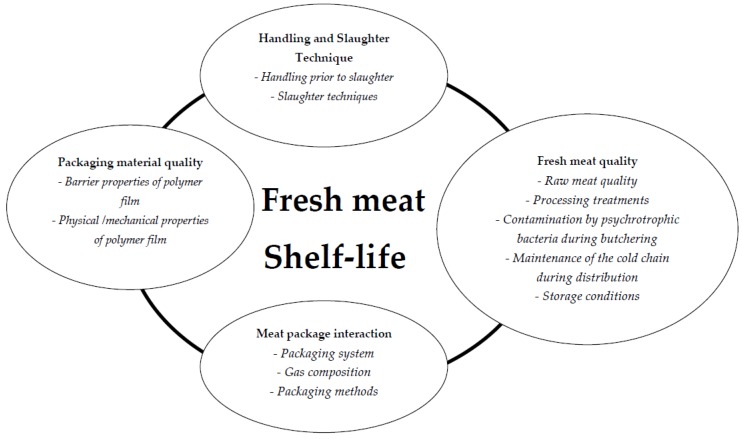
Factors influencing shelf-life of fresh meat.

Maintaining quality attributes throughout its shelf-life has been a perennial challenge for the meat industry and government agencies (Figure 2). Shelf-life is a frequently-used term that can be understood and interpreted differently. In 1974, the U.S. Institute of Food Technologists defined shelf-life as “the period between the manufacture and the retail purchase of a food product, during which the product is in a state of satisfactory quality in terms of nutritional value, taste, texture and appearance”. According to The Institute of Food Science and Technology in the United Kingdom, shelf-life is “the period during which the food product will remain safe; be certain to retain desired sensory, chemical, physical, microbiological and functional characteristics; and comply with any label declaration of nutritional data when stored under the recommended conditions”.

In the EU, shelf-life is not defined in law, nor is there legislation about how shelf-life should be determined. According to EU regulations (Directive 2001/95/EC of the European Parliament and of the Council on General Product Safety), the manufacturer is responsible for putting safe products on the market. More recently, fresh beef shelf-life was determined based on sensory analysis, chemical, physical and microbiological properties. Shelf-life was therefore defined as “the period between slaughter of animal and the simulated retail purchasing, during which the meat retains all its qualities attributes” [18]. However, in commercial practice, this definition overlooks the fact that the consumer may store the product at home for some time before consumption. 

### 2.3. Packaging Options

The role of centralized packaging systems in the longer modern food supply chains is being increasingly recognized as it has multiple functions and is very important in terms of increasing product shelf-life by retarding food-quality degradation and ensuring food safety [40]. The four major packaging systems include vacuum packaging (VP), vacuum skin packaging (VSP), modified atmosphere packaging (MAP) and polyvinyl chloride over-wrap film (PVC). The four approaches differ in their preservative capabilities and their applicability to the centralized packaging of retail meat. Moreover, fresh meat commercialization strategies have notably changed during the last few decades. Therefore, maintaining quality and color appearance is fundamental during the distribution and marketing of meat. For this reason, packaging innovation has become indispensable to increase the shelf-life of this product.

#### 2.3.1. Emergence of MAP Case-Ready Meat Products

During the last decade, case-ready meat packaging techniques continued to grow. Traditionally in the industrialized countries, fresh meat is wrapped in oxygen-permeable polyvinyl chloride film. The advantage of this technique allows rapid oxygenation of the surface pigments and development of the red bright color, but oxidation of pigment occurs within a few days (1–7 days). Over the past decade, the use of case-ready meats has increased in the U.S. and developed countries’ markets. However, as a result of an increasing demand for fresh and ready-to-use products, a need has emerged for further studies involving the possibility of extending the shelf-life of the refrigerated meat. In fresh meat, Mb can exist in several redox states. Understanding pigment chemistry has promoted central packaging of case-ready meat products, which can increase color shelf-life via the use of MAP and other technologies. These products are cut into consumer-ready portions, packaged and typically sold under labeled names that consumers can accept or reject when making purchasing decisions.

Commercial retailing of fresh meat packaged under modified atmosphere (MA) tray systems was introduced in the early 1970s. Case-ready modified atmosphere packaging (MAP) can reduce the costs of fabrication and packaging at retail outlets and other benefits; like preventing from being out-of-stock. The most common gas mixture used for MAP fresh beef is 80% O_2_ and 20% CO_2_. High O_2_ packaging could increase color stability up to 21 days [19] compared to 4–7 days for meat packaged in O_2_-permeable PVC film over-wrap [20]. However, at purchase point, meat initial microbiological load, meat temperature history, intensity of light, display period, temperature, location in the display case and characteristics of packaging materials frequently determine the effectiveness of preservative packaging.

#### 2.3.2. High O_2_ MAP

MAP is based on modifying the composition of gas in contact with food by replacing the air with a single gas or a mixture of gases (gas naturally present in the atmosphere). The major gases in dry air by volume at sea level are N_2_ (78%), O_2_ (20.99%), argon (0.94%) and CO_2_ (0.03%), but the percentages vary when calculated by weight [20]. The use of MA is not a new concept in food preservation. In the 19th Century, scientists discovered that high levels of CO_2_ showed antimicrobial effects. Since the 1930s, the Australian meat industry was using CO_2_ atmospheres to extend shelf-life of fresh meat exports, but for a lack of auxiliary means, MAP processing was replaced with freezing after World War II due to lower costs and longer shelf-life. During the last few decades, HiO_2_-MAP has been and continues to be widely used in case-ready meat production. HiO_2_-MAP promotes the desirable bright red color of meat during storage and display due to Mb oxygenation. The most common gas mixture is 60–80% O_2_ and 20–30% CO_2_ [41], even though it is demonstrated that a minimum of 55% O_2_ is sufficient to maintain a good meat color [2]. Even though a limiting value of about 5% O_2_ partial pressure is needed to maintain O_2_Mb [22], O_2_ higher than 13% will provide predominant O_2_Mb pigments [42]. This is readily achievable with air-permeable overwrap packaging or HiO_2_-MAP. 

##### Gases Used in MAP

The conventional gases used in MAP are N_2_, O_2_ and CO_2_. The choice of gas used singly or in combination depends on the packaged product. Today, packaging films are available with different gas permeabilities to meet the different requirements of the food industry. CO_2_ is present in the atmosphere at a low level (0.03%). CO_2_ is a colorless gas with a slight pungent odor at high levels. The solubility of CO_2_ in water and lipid phases of the product increases with decreasing temperature [43]. For this reason, the antimicrobial activity of CO_2_ is markedly greater at temperatures below 7 °C. This has significant implications for MAP of foods. 

The antibacterial effects of CO_2_ have been known for decades. CO_2_ is effective against psychrotroph bacteria and has high potential for shelf-life extending of chill-stored food [44]. Gram-negative bacteria were considered more sensitive to CO_2_ than Gram-positive bacteria. However, various authors indicate that high levels of CO_2_ (>20%) result in undesirable brown color in meats [45]. The antimicrobial mechanism of CO_2_ could be explained by its ability to penetrate the bacterial membrane, causing a great intracellular pH change [46]. In general, CO_2_ increases the lag phase and generation time of spoilage microorganisms. Antibacterial effects of CO_2_ are markedly temperature dependent, and it is therefore imperative that chill temperature be maintained across the supply chain for health concerns, thus enhancing the shelf-life of the perishable meats. O_2_ is a colorless, odorless gas that is highly reactive and supports combustion. It has a low solubility in water. O_2_ promotes several types of deteriorative reactions in foods including fat oxidation, browning reactions and pigment oxidation. Most of the common spoilage bacteria and fungi require O_2_ for growth. HiO_2_-MAP has been and continues to be widely used in case-ready meat production. HiO_2_-MAP promotes the desirable bright red color of meat during storage and display and can slow pigment oxidation. However, HiO_2_-MAP can increase lipid and protein oxidation and supports the growth of spoilage bacteria with negative effects on meat flavor and texture [47]. Another problem created by exposing beef to high O_2_ atmospheres is called “premature browning”. N_2_ is a relatively un-reactive gas with no odor, taste or color. The low solubility of N_2_ in foods can be used to prevent pack collapse by including sufficient N_2_ in the gas mix to balance the volume decrease due to CO_2_ and O_2_ absorption into the product. It is used to displace air and, in particular, O_2_ from MAP. Since air and consequently O_2_ have been removed, the growth of aerobic spoilage organisms and oxidation reactions are inhibited or stopped. N_2_ is an inert gas that is not reactive with meat pigments or absorbed by the meat; therefore, it prevents from collapsing and maintains the integrity of the package by its presence in the headspace. The noble gases such helium (He), argon (Ar), xenon (Xe) and neon (Ne) are characterized by their lack of reactivity. These gases are being used in a number of food applications. 

#### 2.3.3. Vacuum Packaging

VP is another common method used to distribute meat and continues to be in many cases the most cost-effective packaging strategy. VP extends the shelf-life of beef even longer than HiO_2_-MAP. Although residual O_2_ levels of 0.15–2.0% predispose fresh beef products to browning [13] because fresh meat is very susceptible to MetMb formation by low O_2_ pressure, it may be necessary to reduce residual O_2_ to 0.05% or lower to inhibit MetMb formation and induce optimal re-blooming upon exposure of meat to air. Various O_2_ absorbers or scavengers have been used to reduce the concentration of residual O_2_. The main desirable effects of VP are inhibition of off-odors [48] and spoilage by *Pseudomonas*, but generally, consumers prefer meat with a bright red color compared to the darker VP beef [15]. Another disadvantage of VP is the purging of the vacuum pack in the folds after air removal, which often leads to increased microbial growth and less attractiveness to consumers [49]. The determination of the barrier properties of a polymer is crucial to estimate and predict the product-package shelf-life during storage and subsequent display of vacuum-packaged fresh beef. The specific barrier requirement of the package system is related to the meat product characteristics. 

Vacuum skin packaging (VSP) is an alternative to conventional VP for retail portions. VSP is an advanced type of VP, which helps to avoid the formation of film wrinkles by making the upper film shrink tightly around the meat, and consequently, meat in VSP is considered more attractive. VSP is a technology that has been designed to prevent purge loss while maintaining many of the benefits of VP. In recent years, this packaging technology has grown significantly in industrialized countries due to the demand for a more tender meat with a long shelf-life during retail/display. The O_2_ deficiency in the VSP case has the same effects as those of VP; the meat in this case has a dark purple color due to DeoxMb [50]. In recent years, meat marketing efforts have been carried out by large meat industries and large supermarkets to attract a wide range of consumers with respect to the individual cuts of vacuum-packed meat; surprisingly, this approach has not been successful [51]. 

#### 2.3.4. Safety of MAP

Maintaining the quality and safety of muscle foods from slaughter to consumption is highly important in the modern food supply. Concerns have been expressed about the risk of pathogens in meat packaging under MA. The increase in shelf-life of MAP meats through inhibition of spoilage bacteria may provide sufficient time for stimulated pathogens to grow to dangerous levels while the food still remains attractive to the consumer. Whatever the packaging system, keeping the continuous chill chain throughout all of the storage period is the most important key factor influencing the storage life of fresh meat. MAP of fresh meats is generally considered less hazardous if cooking is correctly carried out. It is also imperative that catering factories and consumers at home maintain adequate refrigeration and, if it is possible, monitor internal temperature during cooking to assure that products reach a high internal temperature to assure destruction of pathogens.

The hurdle concept is widely accepted as a food preservation strategy; its potential, using MAP, has still to be fully realized. When MAP is combined with other preservation methods, its effectiveness may be highly enhanced [52,53]. 

## 3. CO in Fresh Meat Packaging

### 3.1. What Is Carbon Monoxide?

CO is a toxic gas. It is produced by the incomplete combustion of carbon-based materials (fossil fuels, industrial and biological processes, wood, etc.) [11,54]. CO is an odorless, colorless, tasteless gas, non-irritating and non-suffocating. Its density is very close to that of air (0.967). It diffuses very quickly in the ambient environment occupying all the space available, which is potentially dangerous in a closed environment. In the biological medium, it is easily bound by coordination to the divalent iron (Fe^2+^) or to the copper (Cu^2+^) of the hemoproteins. In addition to its production by the incomplete combustion of hydrocarbon materials, significant quantities are also produced during the operation of internal combustion engines due to incomplete combustion of the fuel. A small quantity of CO is naturally produced endogenously in humans [55], regulating blood flow and blood fluidity [56]. CO can be produced during irradiation of meat [57,58,59,60,61]. The production of CO in irradiated samples was irradiation-dose dependent [58,62]. The major sources of CO in irradiated meat are amino acids and phospholipids [63]. CO is also produced by reactions between meat components and free radicals produced by radiolysis [58,62,64]. 

### 3.2. Health Implications of CO

CO is called the “silent killer” because if the early signs are ignored, a person may lose consciousness and be unable to escape the danger. The first descriptions of CO and its toxic nature appeared in the literature over 100 years ago; recognized since 1895 by Haldane [65]. The ancient Greeks and Romans used CO to execute criminals. Application of CO for sedation and killing of animals has mainly been carried out in scientific studies, but has been practiced at an industrial level for killing of mink. Use of CO for sedation and killing may be important, especially in order to enhance both the animal welfare and the overall meat quality. Utilization of CO for euthanizing of animals may also be carried out by veterinarians at laboratory facilities [66]. In most countries, stunning prior to sacrificing of animals is required. The method to stunning should ensure that the animals reach an unconscious state fast, without any pain, and the animals should not recover consciousness before death. Meanwhile, Kosher and Halal practices of the slaughter of animals without stunning are in use.

The past twenty-seven years have been marked by an explosion in the number and quality of studies regarding the actions of CO in mammalian systems. The toxic action of CO is due to the blockage of the O_2_-carrying function of hemoglobin (Hb) through the formation of COHb instead of oxyhemoglobin (O_2_Hb) and prevents the body from using O_2_. Thus, cells, tissues and vital organs may become hypoxic and undergo irreversible anatomical, biochemical and physiological changes, leading to death and morbidity. Thus, symptoms appear when COHb is >10% [67]. The fetus and infant are the most predisposed to harmful effects of CO compared to adults due to higher metabolism and the presence of fetal hemoglobin, which has a greater affinity for CO than adult hemoglobin [68]. On the other hand, the risk of developing autism in children is also linked to CO exposure [69]. The affinity of hemoglobin for CO is 240-times greater than that for O_2_. A small amount of CO (~0.5%) is formed naturally in the human body from the breakdown of hemoproteins [56]. The average COHb level in nonsmokers is 1.2–1.5% (from both endogenous and environmental CO) and 3–4% in smokers [54]. Clinical results showed that children born to women smokers exhibited low intellectual development and poorer performance on cognitive tasks [70]. The half-life of COHb is 4–6 h in the mother, but much longer in the fetus (18–24 h), which accentuates the effects of hypoxia on cerebral functioning and explains the rates still being higher COHb in the fetus than in the mother. Today, we now know that CO has a number of different action sites. More knowledge about the physiological process involving CO is necessary especially after the discovery of the heme protein neuroglobin (Ngb) in the brain of vertebrates [71]. Neuroglobin is thought to act as a reservoir for O_2_ and in that way prolongs the activity of the nervous system. CO reacts with the heme protein Ngb in the brain and might also take part in biological signaling. It is possible that CO has an important function in humans. CO may have a physiological influence on the mind’s functioning since CO acts as a biological signal in regulating the cyclic guanosine monophosphate (GMP) and most likely works as a neuronal messenger [72,73]. It was discovered that CO is produced endogenously as a cellular protectant by nearly every cell in our bodies when they are subjected to situations of oxidative stress or injury [74,75,76]. Recently, CO has emerged as a potential therapeutic agent for the treatment of various cardiovascular disorders [77,78,79]. CO is a necessary molecule for normal cell signaling and can play a therapeutic role in humans [80]. Kim et al. [81], Onyiah et al. [82], Soni et al. [79] and Steiger et al. [83] established that CO can be used as a potential pharmacological cytoprotective (anti-inflammatory protection) agent against several diseases.

### 3.3. CO Application in Meat Packaging

The use of CO for meat packaging is not allowed in most countries due to the potential toxic effect, and its use is controversial in some countries. Nevertheless, according to Sørheim et al. [16,54], the method of adding 0.4% CO to a commercial gas blend with 60% CO_2_ and 39.6% N_2_ for case-ready packaging systems of beef, pork and lamb was developed by the Norwegian meat industry, starting in 1985. The use of CO for meat was discontinued in July 2004 due to pressure from European trading partners [84]. In Norway, the low CO packaging process grew to 60% of the retail red meat market [85]. The commercial success and safety record of the Norwegian process was a factor in the renewed interest in fresh meat packaging using CO in the United States. CO has a long history of application within the meat industry for its color-stabilizing effect coupled with its antioxidant abilities. Most CO-modified atmospheres contain no O_2_, which limits the oxidation and growth of aerobic microorganisms. CO binds to the sixth coordinate of the heme group centrally located within Mb and forms a bright cherry-red color (COMb). The affinity of DeoxMb for CO is 28–51 times greater than for O_2_ [86]. MbCO is more stable than MbO_2_, making it is less likely to oxidize to the brown pigment, MetMb, during display [87]. Important findings in extending the shelf-life of fresh meats by MAP alone and by other treatments since 2000 are summarized in Table 1.

The use of CO gives promising results in the primary package of fresh meat due to its positive effects on shelf-life prolongation during wider distribution of case-ready products. This, in turn, would reduce product and economic losses. By using CO in a modified atmosphere, the need for O_2_ to achieve a bright color is eliminated, thus the opportunity to eliminate the detrimental product effects that O_2_ imparts to the product. El-Badawi et al. [87] published for the first time an article on the use of CO (air + 2% CO) for the packaging of meat. Previous work reported that MAP with 0.4% CO and VP were the most stable packaging systems for ground beef containing 10–30% fat levels [114]. Modified atmosphere packaging with 0.4% CO is recommended for extended storage of fresh meat in a master-pack arrangement such that export to distant markets can be accommodated [85]. The inclusion of CO in MAP is controversial because the stable cherry-color can last beyond the microbial shelf-life of the meat and thus mask spoilage [23].

#### 3.3.1. Color Stabilizing and Shelf-Life Effects

**Figure 3:**
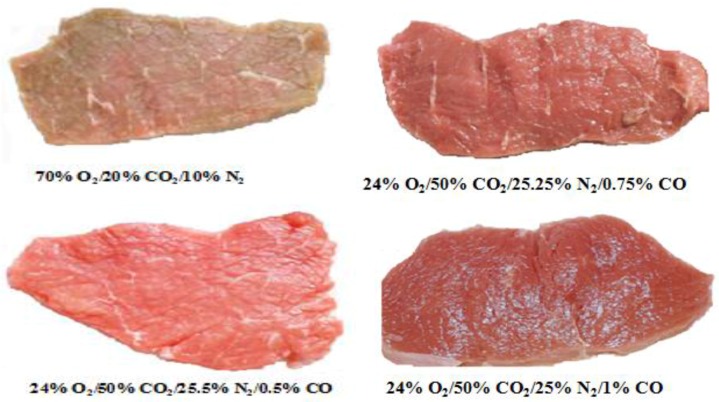
Photos of beefsteaks corresponding to the 21 days of storage under modified atmospheres packaging (MAP) at 1 ± 1 °C [188].

Fresh meat is a highly perishable product due to its biological composition. Meat color is frequently used as an indicator of freshness and wholesomeness, so color plays a critical role in determining consumers’ purchasing decisions. Pigment oxidation, and the subsequent browning, is the primary basis for consumer rejection of fresh retail beef (Figure 3). Considerable effort has been deployed by the meat industry to enhance color stability by innovative techniques. The color of meat depends on the concentration of O_2_ and the oxidation state of the Mb. The shelf-life of meat is limited by the initial MbO_2_ layers formed during the “bloom”, the time required for oxidation of MbO_2_ to MetMb, and reaches proportions of total MetMb concentrations such that the meat appears dull and eventually brown [126,127]. The MbO_2_ is gradually oxidized to form MetMb, and the kinetics of the process is dictated by several factors such as the muscle type, rate of post-mortem pH decline, packaging film, O_2_ consumption, display lighting and temperature and the intrinsic MetMb reducing activity of the muscle [128].

Incorporation of CO in the gas mixture can provide a stable, cherry red color to the meat by formation of MbCO, which is more resistant to oxidation compared to the MbO_2_ [54,88]. Jayasingh et al. [90] studied the penetration of CO and development of COMb layers in whole beef muscle and ground beef depending on exposure time. In a gas blend with 0.5% CO, layers of 2-mm COMb developed in beef muscles after 10 h of exposure. Under the same CO exposure, layers in ground beef were deeper, between 7 and 11 mm. Woodruff and Silliker [129] reported that a concentration of 10% CO can penetrate 0.63–0.94 cm beneath the surface of meat, forming a bright stable red COMb layer. Raines and Hunt [107] studied the effects of headspace volume and CO% on COMb layer development in packaged beef steaks and found that the concentration of CO in a smaller headspace resulted in a thicker COMb layer compared with lesser concentration of CO in a larger headspace. Maintaining meat with an attractive bright cherry-red color during retail display is a challenge for processors and the retail industry. Numerous studies have reported the effects of CO on extended color stability, and additional benefits of the application of CO have also been reported. For its enhancement of meat quality attributes and color stabilizing effects, CO has many value-added benefits in meat packaging. El-Badawi et al. [87] conducted one of the first experiments using CO in packaging of fresh beef (2% CO and 98% air) and found that red color was stabilized and maintained for 15 days at 2–3 °C. Seven years later, the same findings were confirmed by Clydesdale and Francis [130]. Jayasingh et al. [90] reported that steaks and ground beef maintained red color for eight weeks when packaged under CO. The possible reason that CO-MAP could enhance red color stability was related to the higher stability of the COMb than O_2_Mb [12,13], but other potential mechanisms have not been explored yet. Krause et al. [92] found that CIE a* values (redness) of pork chops were significantly higher in CO (0.4%) atmosphere than in those in traditional aerobic packages and that redness was retained throughout 36 days of storage. Luño et al. [88] reported similar results in which ground beef and beef loin steaks packaged in MAP containing <1% CO retained a stable red color for 29 days. The same authors found that the addition of CO to the MAP gas mixture, either in place of or along with O_2_, results in the formation of stable, bright red COMb, and under these conditions, the presence of O_2_ is not problematic to meat color probably since CO is able to enhance Mb reduction even in the presence of O_2_ (the bright red O_2_Mb pigment, arising from exposure of meat to air or high O_2_ atmospheres, is known to destabilize during storage) [11,14] and because the affinity of Mb for CO is 30–50-times greater than its affinity for O_2_ [131]. Additionally, Liu et al. [115] reported that 0.4% CO-MAP packaging systems can maintain higher MetMb reducing activity (MRA), which is linked to increased color stability, compared to high-O_2_ MAP. In contrast, the addition of 0.4% CO to either 20% or 80% O_2_ atmospheres did not increase the MetMb reducing activity in five beef muscles [132]. Recently, Sakowska et al. [124] found that CO-MAP packaging systems significantly increased redness of beef steaks during storage for 21 days. Sausages packaged under CO discolored faster during air and light display than sausages treated with nitrite. However, discoloration of CO-packaged sausages was reduced by anaerobic storage in darkness, showing that absence of O_2_ is a necessity for optimum color stability of these products [97]. Previous studies have proved that CO can significantly increase color stability of beef compared with other packaging methods. Steaks in 0.4% CO within a master bag achieved 21 days of desirable red color compared with steaks in 80% O_2_, which had the highest TBA values [94]. The color stability of beef strip steaks packaged in a 0.4% CO anoxic atmosphere is greater than the color stability of beef strip steaks packaged in HiO_2_-MAP [98]. More consistent color stability of beef steaks was obtained by 0.4% CO use (35% CO_2_/69.6% N_2_) when compared with HiO_2_-MAP (80% O_2_/20% CO_2_) [105]. However, when 1% CO-treated ground beef was exposed to air, the bright red color was lost within a few days [44,133]. Jeong and Claus [108] also found that 0.4% CO-packaged ground beef became less red after opening the package compared with the color of ground beef packaged in VP. However, the factors affecting color loss of beef steaks in CO-MAP upon opening the packages are still not fully understood. Additionally, the variable color stability of CO-packaged meats from different muscles after opening the packages is not clear. The phenomenon of premature browning was evident during cooking of steaks stored in high O_2_ [94]. Raw ground beef held in 0.4% CO remained bright red throughout the 21-day storage period. Premature browning in cooked patties was avoided by use of this packaging system [93]. Beef that was treated with 100% CO/3 h before freezing, maintained a bright red color during frozen storage for 90 days [134].

Meat products are commonly manufactured with the addition of nitrite or nitrate. An important function of these ingredients is to create a stable red to pink color of these products. The disadvantage of nitrite is the formation of carcinogenic nitrosamines in nitrite-treated meat products and in vivo by consumption of the products [135]. Sørheim et al. [97] found that CO can be used as an alternative colorant to nitrite in these products. A gas mixture containing 1% CO was sufficient for achieving a red/pink color. Low CO packaging also improves the color life of irradiated ground beef to complement the greatly improved microbial shelf-life achieved by the irradiation process [91]. The stability of meat color is closely related to intrinsic factors of muscle type and metabolism and extrinsic factors of packaging type [136,137]. Each muscle has a unique fiber type and metabolic function. Color stability is also affected by the inherent O_2_ consumption rates, oxidation-reduction potential, MetMb reducing capacity and MetMb reductase activity of muscles [136,137,138].

The shelf-life of perishable meats is limited in the presence of normal air by two principal factors: the chemical effect of atmospheric O_2_ and the growth of aerobic spoilage microorganisms. Chilled storage will slow down these undesirable factors, but will not necessarily extend the shelf-life sufficiently for retail distribution and display purposes. Studies were carried out to evaluate the effect of high CO_2_/low CO MAP on the shelf-life of fresh meat and meat products under MAP conditions [16,54]. The shelf-life of pork chops was extended to more than 36 days in CO-MAP compared with only 28 days in traditional, HiO_2_-MAP, 23 days in VP and 7 days for overwrapped (OW) packages [92]. The shelf-lives at 4 °C during storage of retail-ready meat in high CO_2_/low CO mixtures of ground beef, beef loin and pork chops were 11, 14 and 21 days, respectively, when stored in 0.4% CO, 60% CO_2_ and 40% N_2_ [16]. It has been concluded that storage in low CO, high CO_2_ atmospheres is effective for extending the storage shelf-life of cuts. The combination of CO and CO_2_ in MAP was beneficial in extending the shelf-life of fresh pork sausage [139]. The use of this gas mixture in fresh meat packaging gives promising results due to its positive effects on color and microorganism growth inhibition, which result in the shelf-life extending during the wider distribution of case-ready products [140]. 

#### 3.3.2. Antimicrobial Effects

In CO-MAP of meat, the effects of low concentrations of CO on microorganisms seem to be of either no or minor importance. CO has been reported to prevent the growth of microorganisms [141]. Combinations of CO with other gases such as CO_2_ to control microbial growth provide an excellent opportunity for meat processors to improve shelf-lives of the retail packed fresh meats [23,142]. CO is selectively bacteriostatic for various microbial populations. CO extends the lag phase and slows the growth rate of *Escherichia coli*, *Achromobacter* and *P. fluorescence* [143] at concentrations of 25–30%, while *P*. *aeruginosa* is unaffected even at these high concentrations. Gee and Brown [44] conducted research using CO and CO_2_ together in an MAP system. Beef patties exposed to 1% CO, 50% CO_2_, and 49% air had two log lower levels of bacteria/g than controls after six days of storage. Additionally, Clark et al. [144] showed that increasing CO concentrations with the balance gas being N_2_ on beef steaks inhibited the growth of psychotropic bacteria, which also had a positive effect on increased odor shelf-life. This result was due to CO having the ability to increase the lag phase and to reduce the log phase. Luño et al. [88] showed that CO-MAP greatly reduced the psychrotrophic bacteria populations including *B*. *thermosphacta* in beef, although lactic acid bacteria appeared to be unaffected. Viana et al. [145] evaluated pork loins packaged in 99% CO_2_ in combination with 1% CO and reported that *Pseudomonas* sp. growth was limited, and psychrotrophic organisms did not reach 10^7^ cfu/g until after Day 20. In CO-MAP, meat maintains bacterial levels less than spoilage levels (~7 log_10_ cfu/g) for ~1 month, but acceptable red appearance is maintained for at least eight weeks [90,146]. This emphasizes the importance of the “use or freeze by” dating system established by the USDA for retail sale of meat in CO-MAP. Woodruff and Silliker [129] reported that a concentration of 10% CO can inhibit microbial growth, further preventing odor and slime by-products. Gee and Brown [14] investigated the effects of different concentrations of CO on pure bacterial cultures of *Pseudomonas*, *Achromobacter* and *E*. *coli* species. It was concluded that 15–30% CO had an inhibitory effect on the growth of bacteria. These levels, however, far exceed the levels legally allowed for use in the packaging of meat products [10].

At 10 °C, CO-MAP has inhibitory effects on *Yersinia enterocolitica*, *Listeria monocytogenes* and *E. coli* O157:H7, but was not as inhibitory against *Salmonella* strains, indicating that chilled storage is important, regardless of packaging method [89]. In a similar experiment, Cornforth and Hunt [11] indicated that CO in MAP inhibited growth of *E*. *coli* O157:H7 on inoculated meat even at abuse temperatures of 10 °C. Contrary to all these observations, Bórnez et al. [147,148] found that the use of low CO (69.3% N_2_/30% CO_2_/0.7% CO) in the packaging of suckling lamb meat did not improve the microbial quality of the packed products. It would be interesting to point out that in realistic concentrations, CO as such has no antimicrobial effect, and CO_2_ in sufficient concentrations is required for delaying the growth of Gram-negative bacteria.

#### 3.3.3. Other Effects

Meat tenderness is one of the most commonly-used parameters for the evaluation of meat quality by consumers. The tenderization of meat is reduced under HiO_2_-MAP because of protein oxidation [149]. Grobbel et al. [103] demonstrated that beef stored in vacuum or anaerobic 0.4% CO atmospheres was tenderer than in HiO_2_-atmospheres. Reduced tenderness is one of the major detrimental effects of the commonly-used HiO_2_-MAP. Thus, meat texture is a highly important factor for the meat industry to consider during the production of meat. Cross-linking of myofibrillar proteins is believed to reduce meat tenderness by causing a strengthening of the myofibrillar structure in meat stored under HiO_2_-MAP. There have been conflicting findings toward the effects of irradiation on meat characteristics [150,151,152]. It is well known that irradiation can produce safe foods and extend shelf-life by eliminating food-borne pathogens, as well as spoilage microbes. However, irradiation doses required to kill pathogens can cause undesirable changes in meat color, flavor and odor. Kusmider et al. [91] and Ramamoorthi et al. [104,111] suggest that CO-MAP could be used to preserve beef color irradiated at doses sufficient to reduce microbial loads to safe levels during 28 days of storage, thus countering the potentially negative color effects of irradiation. Lactate is a commonly-used injection-enhancement ingredient that stabilizes the color of beef products by minimizing surface color change through the production of a dark pigment that remains stable during retail storage and display [153]. The ability of CO to produce a bright cherry-red color may counteract lactate’s darkening effect. Premature browning is a condition in cooked meat in which the inner parts of the meat turn gray/brown and appear well done at a lower temperature than expected around 60 °C, thus causing the risk of consumption of undercooked meat with pathogenic bacteria. The condition is associated with exposure to O_2_ and formation of MbO_2_ in the raw meat. Packaging in vacuum or atmospheres containing 0.4% CO without O_2_ prevented premature browning, both in ground and whole muscle beef [94], as well as enhanced beef [103]. For bone-in meat, the marrow with its content of Hb is sensitive to pigment oxidation. Storage of bone-in beef in an anaerobic atmosphere with 0.4% CO prevented marrow browning [13]. To minimize darkening problems due to the use of blood in food formulations, various solutions have been proposed. In a previous attempt to solve this problem, Fontes et al. [106,119] showed that blood saturated with CO produced a pigment with a stable and desirable color that could allow a greater amount of blood addition to meat products that would not lead to their browning. As a replacement for nitrite, a 1% CO gas blend was used for the storage of meat raw materials for dry cured sausage or flushed through sausage batters that later were cooked to 80 °C [97]. The sausage had a red color after production, equal or more intense than with nitrite, but the color faded upon air exposure. 

#### 3.3.4. CO in Other Foods

##### Fruits and Vegetables

The USA have applied CO within vegetable processing since the 1970s to prolong the shelf-life of iceberg lettuce during distribution [154,155,156], and it is recommended as a component of modified atmospheres to prolong shelf-life of tomatoes, cauliflower, cantaloupe, strawberries and citrus [46]. CO has been reported to prevent the growth of fungi in various vegetable foods. Kader [155] showed that CO-MAP greatly reduced the *Botrytis* rot on tomatoes, strawberries and grapes and brown rot on peaches. CO also has been included in the MA of marine transport of foods [155], in part to kill mites and other insects [157]. Incorporation of CO in the gas mixture with a slight vacuum can provide a stable color in minimally-processed fruits and vegetables [158].

Low CO-MAP significantly delayed the internal browning and softening during chilled storage of peach fruit, and the synergistic effects were recorded in combination with 1% of chitosan [159]. Recent research suggested that CO could enhance the anti-senescence ability of plant leaf, improve the superoxide dismutase (SOD), peroxidase (POD) and catalase (CAT) activities of plant tissue and reduce the MDA content of fresh cut Chinese rose flower. In addition, CO fumigation could prevent browning and maintain quality of fresh-cut lotus root slices. The inhibiting browning by CO was related to reducing the activities of polyphenol oxidase (PPO) and POD [160]. A grain storage loss is caused by insect infestation. The application of CO could be used to increase the efficiency of CO_2_, especially at high temperatures [161].

##### Seafood and Poultry

The food industry is continuously attempting to develop new technologies aimed at extending the shelf-life of fish products, without modifying their nutritional and sensory attributes. A bright red color is an important quality determinant in seafood, particularly tuna, as the market value is based on this attribute [162]. Tasteless filtered smoke has been used in salmon smoke houses for decades. Some taste and odor components like phenols, carcinogenic compounds and gases are removed by passing the smoke through washing and several filtration steps. The tasteless smoke naturally contains 15–40% CO [163].

The use of CO either alone or as part of a filtered wood smoke (FS) process has been applied to seafood to maintain the desirable and stable color attributes during storage and distribution [112]. In the USA, CO-MAP, up to 0.4%, are used commercially for packaging of meat, while FS containing 30–40% CO is permitted for pre-treatment of fish [97,164,165]. Fillets of tilapia (*Oreochromis niloticus*) were treated with 100% CO for 30 min, vacuum packaged, frozen at −20 °C and thawed. The CO pretreatment increased the redness and microbiological stability of the fillets, while it did not affect pH, drip or thaw loss [166]. CO is widely used as a supplement for ice or refrigeration storage to delay spoilage and extend the shelf-life of fresh fishery products [167]. Exposure of salmon (*Salmo salar*) or other fish to CO could improve quality and welfare when slaughtered [109,116]. 

It is very important to assess the toxicological risk associated with the extended shelf-life of muscle foods. Seafood products are of high concern; in particular, for histidine-rich fishes, such as tuna, mackerel, sardine, herring and swordfish, the fraudulent use of CO provides an additional risk since histamine (oxidative decarboxylation of histidine), responsible for toxicological effects, can be formed. The color of turkey meat benefitted from the addition of 0.5% CO [168]; but chicken meat contains low levels of Mb, and the color is less affected by CO.

#### 3.3.5. Co Pre-Treatments

CO pre-treatments before VP can be a good alternative for meat retailers. Additionally, the application of CO pre-treatments prior to VP may overcome this issue, as CO is not present in the pack during storage, and therefore, the risk will be minimized when the trays are opened at the household level. Moreover, the addition of CO pre-treatments prior to VP may be beneficial to allow a desirable color to be induced while allowing aging to occur within the package and increasing meat tenderness. Many studies have reported pre-treatment of meat with CO [92,121,123]. In a study of chilled beef steaks, a pre-treatment with CO stabilized the red color of vacuum sealed meat for 4–8 weeks [169]. In a similar experiment, CO pretreatment, followed by VP and chilled storage of beef steaks, gave an initially more red color, but after six weeks, the redness was similar to that of untreated meat [12]; and the aerobic plate counts were lowered by one log after eight weeks of vacuum storage, compared to no CO pre-treatment. A study carried out by Jayasingh et al. [90] investigated the color stability of beef steaks exposed to pre-treatments of CO prior to VP and chilled storage, aiming at synchronizing microbiological and color shelf-life. The 5% CO pre-treatments prolonged the color shelf-life for five weeks, whereas the 100% CO pre-treatment maintained a color shelf-life of six weeks. They concluded that a pre-treatment for 24 h in a 5% CO-MAP was needed to maintain redness after re-packaging in VP for >21 days and was practical for large-scale application. A 5% CO, 95% N_2_ pre-treatment of vacuum-packed beef markedly enhanced the red color and is likely to increase its acceptance by the consumer [99]. The only inconvenience arising from this technique lies in the following fact: the red color of CO-treated meats gradually changed to a brownish discoloration when cuts were stored under aerobic conditions [144]. In contrast, meats continuously held in the presence of low CO/high CO_2_ in an MAP environment maintain red color for extended periods. The barrier properties of the films used for the packaging of food are crucial. Therefore, knowledge of gas transfer rates through the material is valuable information.

## 4. Consumer’s Perceptions

At the point of purchase of meat, color, price and visible fat are considered key factors, while tenderness, flavor and juiciness are more closely related to meat eating satisfaction. On the choice of consumers, because it is most difficult to evaluate before purchase, and it is not visible and highly variable. The confidence of consumers in meat based only on color as an indicator of spoilage has probably been exaggerated. Although, at the retail level, consumers will purchase brown meat at a discount, which means that color alone does not indicate the degree of freshness related to meat, a study by Carpenter et al. [170] showed that eating satisfaction of cooked beef was unaffected by the color of the raw meat during the purchase. It is recognized that brown color of raw beef was not a definitive indicator of spoilage. 

During the last few years, the use of CO in the food industry has been debated strongly and seriously by several public and private organisms. Cornforth and Hunt [11] concluded that the major disadvantages of CO-MAP of red meat were the negative image of CO held by consumers because of its potential toxicity; on the other hand, storage of products under inappropriate conditions and in the presence of CO could potentially mask visual evidence of microbiological spoilage. It is believed that this stable red color may conceal microbiological spoilage and place consumers at risk. However, Hunt et al. [146] and Brooks et al. [171] showed that addition of 0.4% CO to modified atmospheres for chilled beef did not mask spoilage, even when the color stability was increased. Some recent studies indicated that consumer decisions to eat meat are gradually becoming more influenced by nutrition and health considerations. A consumer preference study that was carried out in Denmark, Norway and Sweden demonstrated that Scandinavian consumers preferred beef steaks in low CO-MAP (0.4% CO) [172]. In contrast, high O_2_ packaged steaks were described as more well done, as an effect of premature browning during cooking [173]. Currently, consumers are not informed by the package itself regarding use of CO or elevated O_2_ levels in the headspace of MAP meats. Due to the lack of consumer understanding of the science and being misinformed about this technology, consequently, to improve consumer attitudes about CO packaging of fresh meat, communications should be designed to not only inform consumers about the use of CO, but also familiarize consumers with the science of this technology. Grebitus et al. [174] have conducted a study on the preferences of U.S. and German consumers towards the ground beef qualities enhanced by CO-MAP. Results show that consumers in both countries have clear preferences for shelf-life extension expressed by cherry red meat color. U.S. consumer’s preferred longer shelf-life as long as the technology is understandable. However, when information is provided about the use of CO in meat packaging, the willingness of U.S. consumers to purchase decreases, and the willingness of some German consumers to purchase increases. Therefore, it is clear that preferences are heterogeneous towards cherry red color once consumers have been previously informed about CO-MAP technology.

An increase in personal knowledge and media exposure influenced acceptance of CO-MAP negatively. Such information can benefit the meat industry, which makes decisions about investing in new CO-MAP packaging methods. Countries differ not only with respect to regulations, but also with regard to consumers’ attitudes towards new technologies. Similarly, two recent consumer studies were carried out to evaluate whether Polish consumers would accept CO in meat packaging systems. Consumers had a preference and increased desire to purchase steaks packaged after low CO-MAP pre-treatments for its attractive cherry red color. Consumers did not accept untreated vacuum-packaged beef steaks as they were considered the least attractive and desirable [121]. Therefore, the results from these studies show promise for the future potential application of CO within the EU, despite the current EU prohibition of MAP with CO. 

Even more, a high percentage of consumers have shown their reticence toward a fresh meat that even though red in color was beyond its use-by date and had a noticeable off-odor when opened at home. 

These findings claim that low CO-MAP is not misleading and that the use-by dates on the packages, coupled with sensory attributes, all contribute to the decision making by consumers about when to reject or accept the product, having shown their reticence towards meat that, although red, has passed its expiration date and has a noticeable odor when the meat container was opened in the home’s kitchen. These results may reinforce the hypothesis that CO use is not misleading and that expiry dates on packaging, together with other sensory factors, contribute to consumer decision making when preparing the product for the first time. When this kind of meat is handled and cooked properly, they are also safe.

## 5. Analysis of CO in Food

A variety of methods is available for the analysis of CO in food materials and in blood, including spectrophotometric methods, infrared analysis and gas chromatography. Analysis of CO can be used to control whether muscle food products have been treated with CO or not, despite that they have not been labeled as such. These considerations underline the suitability of this method to detect even small amounts of the CO-Mb adduct in fish and meat tissue, in regard to the fraudulent treatment of muscle foods in the MAP system. The official method for quantification of CO has a problem, in that a part of the CO is lost during the preparation of the food matrix sample [175]. The official laboratories of food control need not only confirmatory methods, but also rapid low cost screening methods for the everyday activity of food control. Due to the potentially harmful effects of CO, there is a need for precise and reliable measurements. The spectrophotometric method (UV-Vis) has been evaluated in terms of its performance criteria by using tuna fish samples. The results have been compared with those obtained using a head space gas chromatographic technique (HS-GC-MS). The CO levels measured in tuna fish samples by UV-Vis are substantially lower than those revealed by HS-GC-MS [176]. A robust and dependable headspace HS-GC-MS method has been developed and evaluated for the determination of CO-treated tuna fish on the basis of its performance [177].

During the last decade, a simple, confirmative method for quantitative determination of CO in commercially-treated tuna and mahi-mahi (*Coryphaena hippurus*) tissues has been reported using gas chromatography/mass spectrometry (GC-MS), following chemical liberation of CO [162]. Gas chromatography equipped with a flame ionization detector (GC/FID) is an efficient technique with low detection limits and high accuracy [100]. The non-dispersive infrared (NDIR) technique measures continuously the level of CO. the NDIR technique is based on the specific absorption of infrared radiation by the CO molecule. CO has an infrared absorption near 4600 nm. NDIR incorporates a gas filter in order to reduce interferences from other gases, operates near atmospheric pressure and detects CO concentration at 0.05 mg/m^3^ [178]. 

Measurement of consumer’s CO exposure level from consumption of CO-packaged meats was based on U.S. Environmental Protection Agency National Ambient Air Quality Standards (EPANAAQS) [11], which presumed that the human metabolism of CO would be equal for digestive processes after consumption of CO packaged meat as the metabolic reactions after CO inhalation. The EPANAAQS for CO is exposure to 9 ppm of CO for 8 h [179], for a typical person inhaling 5 m^3^ air/8 h [11]. Lavieri and Williams [114] found that the maximum level of CO detected in the CO-treated meat was below 9 ppm. Droghetti et al. [176] revealed that the UV-Vis spectrophotometric method detects exclusively CO bound to the iron (Fe) atom of the heme protein and not the CO dissolved in solution; probably, this technique underestimates the amount of total CO present in solution.

## 6. Safety Consideration of CO-Treated Meat

Little information exists in the literature on the consumer’s exposure to CO-packaged meat. The toxicological aspects of CO used in MAP of meat were reviewed by Sørheim et al. [16], and they concluded that, with up to about 0.5% of CO, no human toxicity was likely. Sørheim et al. [54] and Cornfort and Hunt [11] found that consumption of CO-treated meat is not associated with any health risks, and meat from CO-MAP results only in negligible amounts of CO and COHb in humans. The Norwegian Food Control Authority (NFCA) has not registered outbreaks or a higher frequency of sporadic cases of food-borne diseases linked to such products since 1985. The increased red color stability of meats exposed to CO was recognized more than 100 years ago [180]. However, the application of CO in meat packaging was not then considered feasible because of possible environmental hazards for workers. For safety reasons, gas detectors are necessary in environments in which CO is applied in any form. Nowadays, exposure to CO in an industrial setting (meat industry) is associated with minimal risks, both due to good practice at the working facilities and equipment design. Human environmental exposure to CO varies greatly. In the same order of ideas, Sørheim et al. [54] indicated that the max level of COHb is recommended to not exceed 1.5%. A COHb level of less than 5% in human blood is not associated with any harm to healthy individuals, and the half-life of COHb in individuals is approximately 4.5 h. The same authors indicated that during various decades of low CO-MAP in the Norwegian meat industry, its use was not associated with any risks to workers. Furthermore, charcoal meat grill workers are one of the many occupational groups that are subjected to CO exposure. It was found that the average COHb levels for these people exceed the 5% COHb level recommended by WHO and the National Institute for Occupational Safety and Health (NIOSH) [181]. According to Wilkinson et al. [84], given the stable fresh color of CO-treated meat and the lack of inhibition of pathogen growth by CO, there is concern that CO-MAP under certain conditions may pose a food safety risk because consumers could conceivably purchase meat that appeared fresh, but was in fact microbiologically spoiled. For this reason, the use of CO in MAP is not allowed in the U.S. and EU. The European Commission [182] also raised a valid concern that if CO meat products are stored under inappropriate conditions, for example increased temperature, which can occur during mishandling or transportation, the presence of CO may mask visual evidence of spoilage. Van Rooyen et al. [123] have combined the advantages of CO pre-treated steaks followed by VP with the cherry-red color while ensuring that discoloration occurred by the end of the shelf-life to address consumers’ concerns about spoilage being masked. Following this, the authors determined the optimum exposure time with a 5% CO concentration to be 5 h as this allowed discoloration to occur by the end of a 28-day display period (2 °C). Nevertheless, the increase in color shelf-life of CO-packaged beef is not drastic enough to prevent the negative effects of abusive storage temperatures and extended storage times. The same authors have studied the interrelationships between discoloration, microbial spoilage and off-odors to determine whether a stable COMb formed in CO-packaged meat would extend color life beyond the point of microbial spoilage. No steaks having acceptable color had spoilage levels of microbes (≥log 7). Thus, COMb formed from 0.4% CO did not mask microbial spoilage. Furthermore, Eilert [183] reported that CO does not mask spoilage. When beef was treated with 1% CO for three days (resulting in about 30% saturation of Mb with CO) and then exposed to air, CO was slowly lost; there was a high loss of CO upon cooking (~85%) of CO-treated meat [184]. Concerning fishery products, there are no direct health implications from eating CO-treated tuna. However, since CO treatment makes tuna (*Thunnus albacares*) appear fresh, in this respect, it has to be considered that tuna fish is most commonly associated with incidents of histamine intoxication. Histamine can be formed by oxidative decarboxylation of histidine. Similarly, Chow and Chu [185] indicated that there is a major effect of heating on the release of CO from Mb in CO-treated tuna. Regardless of the temperature of heating, the release of CO was 58–82%. Most people as per the present lifestyle preserve animal food stocks at one time; they are unaware that they are exposed to CO, which can create health hazards [186]. The CO level detected in frozen food samples was found to be slightly above 1 μg/g. Recently, an attempt to calculate the COMb in a package atmosphere and very interesting findings have emerged concerning CO and consumer safety. Based on the fact that the typical meat packages have a headspace of 1.5 L and the ambient air quality standard for CO inhalation is 9 ppm/8 h, fresh packaged meat stored in low CO-MAP with a 1.5-L headspace could contain 0.4% CO. Opening one CO-MAP container in an average space (150 m^3^) results in an ambient air CO concentration of 0.042 ppm [11]. Assuming that no COMb has developed, opening one CO-MAP container in 0.8% CO with a 0.4-L headspace results in 0.022 ppm CO in ambient air. After 7 days of display, 9100 of the packages with 0.8% CO in a 0.4-L headspace would have to be opened in one room (150 m^3^) to meet the EPA standard of 9 ppm CO. Thus, reducing headspace from 1.5–0.4 L and increasing CO from 0.4–0.8% do not pose a consumer safety risk [107]. For low CO-MAP (0.4% CO) with a 1.5-L headspace, opening of 216 packages for the same area would be required to exceed the EPA standard [187], for a typical person inhaling 5 m^3^ air/8 h. On the other hand, an assumed consumption of 250 g fresh meat/day could therefore release 0.18 mg CO (~0.018% COHb). If there were a 100% transfer of CO from the gut to the blood, only a negligible amount of COHb would be added to the 0.5% COHb, resulting from endogenous CO production, and ~1.0% COHb formed from inhalation of the contaminated atmosphere by a non-smoker [54]. Realistically, one would consume even less CO per meal because it is known that only 15% of bound CO remains with the meat after cooking [185]. Exposure through inhalation of headspace gas on opening a package of meat with a MAP containing 0.3–0.5% CO would equally contribute insignificantly to the COHb in the blood when compared to the other sources of inhalation of CO [54].

## 7. Regulatory Status for the Use of CO in MAP Systems

Since 1985, Norwegian meat industries have used 0.4% CO for packaging of muscle foods [16]. The use of CO is controversial. Some countries approve the application such as the U.S., Canada, Australia and New Zealand, while the EU member states ban it from meat processing. The EU considers packaging gases as additives, and CO is not on the list of such gases. Until 2004, low CO-MAP was in use in Norway. Norway is not a member of the EU. Due to trade agreements within the European Economic Area, EU food regulations must be adopted by Norway. In 2002, the Norwegian meat industry applied for an acceptance of low CO gas blends for meat in the EU. The application was positively evaluated by a scientific committee of the EU commission, but eventually, CO was not included on the positive list of additives. The EU imposed a prohibition of CO as a food gas, valid from 1 July 2004. In contrast, an approval of commercial use of low CO packaging for retail meat was announced in the U.S.: the USDA and FDA jointly approved the use of 0.4% CO in an anaerobic MAP master-bag system along with 30% CO_2_ and the balance as N_2_ during distribution. Further GRAS approval was registered in the U.S. in 2004 [10] for the addition of CO to primary packaging systems, in which the MAP mixture is flushed directly into the meat-containing tray, declaring that CO does not mask spoilage odor during retail meat packaging. New Zealand and Australia also allow low concentrations of CO in centralized packaging systems, and it is considered a processing aid and, therefore, is not required to appear on the product label [82,189]. Similarly, Canada also regulates the application of low CO as a secondary packaging gas under specific conditions in accordance with Health Canada and the Canadian Food Inspection Agency (CFIA) requirements and standards. The European Scientific Committee on Food considered the addition of 0.3–0.5% CO in the MAP system for fresh meat packaging to be safe for human health [183]. However, due to a lack of legal permission, CO is not yet used in the packaging of fresh meat in the European Union. It was recommended to have expiration dates on the label for beef in low CO-MAP; according to Sebranek et al. [190,191], the FDA has examined this issue thoroughly and requires that meat in low CO packaging be labeled with a “use or freeze by” and decided that open date codes for products packed in the CO-MAP system are 35 days following the date of packaging for intact steaks or roasts and 28 days for ground meats. The specific gases do not have to be labeled on the packages. It would be good to point out that meat is subject to natural spoilage processes no matter what type of packaging is used. It is, therefore, important to keep meat at chill temperatures throughout storage, distribution and during retail/display. Consumers should take care to use the meat by the date indicated on the package and practice safe food handling techniques.

## 8. Could the Application of CO for Meat Packaging Be Re-Considered?

The major factor contributing to retail loss by withdrawal, mainly in industrialized countries, is the discrimination of discolored meat that consumers perceive as unhealthy. In order to reduce these losses and support increased consumer demand and expectation of high meat quality, packaging technology innovations are required. The regulatory aspect concerning the use in developed countries of CO in meat packaging has been a major controversy in recent years. Indeed, the European Union has banned its use in the packaging of meat, mainly because of the fear of masking the deterioration of the packaged product, which could easily mislead consumers. In recent years, great inconsistencies have been recorded between developed countries regarding the adoption of regulations for use of CO in the meat industry, which resulted in obstacles to international trade, limiting export opportunities between countries [17]. The main disadvantage of meat CO-packaging is its possible masking of the microbiological spoilage by formation of the stable, bright red color. For a potential antimicrobial effect, a combination of low levels of CO with high CO_2_ concentrations is a better way, and this is important to justify approval by regulatory agencies. Research on pre-treatment with CO followed by VP is promising because meat pre-treatment with CO prior to VP results in improved color while allowing discoloration to occur by the use-by-date, thus addressing safety concerns, assisting in the flexibility in distribution and preventing losses by rejection from the distribution chain. 2002 was the first time that CO was regulated for meat packaging in the U.S. [192]. Two years later, the same federal agency authorized the use of 0.4% CO as recognized as safe (GRAS) in the packaging of meat in case-ready packaging systems [10]. For more than 20 years, Norway has been applying low levels of CO in meat processing systems. However, the Norwegian government suspended its use in 2004 due to the adoption of EU regulations. Australia and New Zealand also regulate low concentrations of CO in centralized packaging systems [189]. Similarly, the Government of Canada also authorized the application of 0.4% CO in red meat packaging. Several studies on the attitude of European consumers regarding the use of CO for meat packaging have reported positive relationships that suggest its future potential within the EU. European consumers preferred beef steaks in low CO-MAP. Despite the current EU prohibition of MAP with CO [124], the decision to ban the use of this gas in the EU should be reconsidered. Sørheim et al. [54] found the application of low concentrations of CO to meat packaging systems to have no toxic effects evident. The European Commission (2001) reported that inhalation of CO-MAP headspace gas containing 0.3–0.5% CO would have no significant effect on the COHb in the blood in comparison to other sources of inhalation of CO [182]. The report also stated that the amount of CO present in fresh meat packaged in low concentrations of CO-MAP is similar to that of the endogenous CO. It is also important to note that during cooking, a considerable amount (~85%) of CO that is bound to COMb and COHb of the packaged meat is lost. In the EU, packaging gases are considered as additives. In order for CO to be approved as an additive within the EU, the following criteria must be met according to Directive 94/34/EEC.

During the last few years, between some European governments within the EU, great efforts have been made to allow the use of CO under controlled and regulated conditions. However, the consensus concerning this fact could take quite a few years for a common agreement. The debates concerning the use of CO in meat packaging have not seriously taken into account the preferences of consumers [17]. CO-packaged meat should be legally labeled for the presence of CO so that consumers can make decisions about their purchases. Furthermore, on the labeling of trays, the consumer must read “the color of the meat does not in any way mean a reliable indicator of freshness”. This is to protect consumers and prevent them from being misled about the freshness of the product since CO can maintain an acceptable color beyond the shelf-life period. The pertinent scientific results must be accompanied with transparent and early communications in an effort to ensure that government, consumers and the media have what they need to make thoughtful decisions in the public’s interest. Facilitation of information can help to develop future policies to ensure consumer protection, and therefore, the debate over the re-evaluation of the use of CO as a protective gas in meat packaging within the EU may be justified. In addition, the application of CO in the EU could be very profitable from the point of view of exports, retail sales in supermarkets, as well as meet consumer quality demands as a value-added technology. In this context, CO-MAP technology has pronounced efficacy to transform the academic research output to industrial application.

## 9. Conclusions

CO can be utilized at different stages of the value chain of fresh meat, in animal slaughter, distribution, pre-treatment before storage, processing and display packaging. The use of CO in fresh meat packaging gives promising results due to its positive effects on overall meat quality; red color is enhanced and lipid oxidation is reduced. In realistic concentrations, CO as such has no antimicrobial effect, and CO_2_ in sufficient concentrations is required for delaying growth of spoilage Gram-negative bacteria, which results in shelf-life prolongation during wider distribution of case-ready products.

The use of CO is especially controversial. Some countries approve the application such as the U.S., while others, as is the case of the EU member states, ban it from food processing due to the potential toxic effect. The commercial application of CO in meat packaging was not then considered feasible because of possible environmental hazards for workers. CO has previously been reported to mask meat spoilage, and this was the primary concern raised for the prohibition as this may mislead consumers. 

Risk of CO toxicity from the packaging process or from consumption of CO-treated meats is negligible. Moreover, the addition of CO pre-treatments prior to VP may be beneficial to allow a desirable color to be induced while allowing aging to occur within the package and increasing meat tenderness. Additionally, CO is not present in the pack during storage. 

Countries differ not only with respect to regulations, but also with regard to consumers’ attitudes towards new technologies. Recent European consumer acceptance studies demonstrate the promising future potential of the application of CO within the EU. Consequently, to improve consumer attitudes about CO packaging of fresh meat, communications among all segments of the meat cold chain should be designed to not only inform consumers about the use of CO, but also familiarize consumers about the science of this technology. In addition, the application of CO in the EU could be very profitable from the point of view of exports, retail sales in supermarkets, as well as meeting consumer quality demands as a value-added technology. Possible problems can arise related to detecting CO treatment of foods. This is a key issue to assure fair competition and to gain consumer’s trust. In light of all the data cited in this article, it can be said that the application of CO for the packaging of fresh meat could be re-evaluated. In this context, CO-MAP technology has pronounced efficacy to transform the academic research output to the meat company’s application.

Recommendations and future prospects the addressed food industries, consumers and regulators on what would be a “best practice” in the use of CO in food packaging:

- The main disadvantage of using CO for meat packaging is the concern about masking of the microbiological risk by the formation of the stable and longer, bright red color.
- A combination of low levels of CO with CO_2_ in high concentrations, which reduce the growth of microorganisms, is of key importance to justify approval by EU regulatory agencies.
- It is also imperative that CO risk management be implemented in the packaging operations. The supermarkets and consumers at home must handle meat with strict hygienic standards, and low storage temperatures must be kept in a continuous chill chain.
- CO followed by vacuum packaging is promising because of the possibility of better adjusting the color stability of the meat to the time of spoilage.
- By adopting CO for meat and fish MAP, the meat industry must label the packages with reliable times for maximum shelf-life (the use-by date).
- The EU considers packaging gases as additives, and CO is not on the list of such gases. In order for CO to be approved as an additive within the EU, the following criteria must be met according to Directive 94/34/EEC. “They present no hazard to the heath of consumer at the level of use proposed, so far as can be judged on the scientific evidence available; to provide aids in manufacture, processing, preparation, treatment, packing, transport or storage of food, provided that the additive is not used to disguise the effects of the use of faulty raw materials or of undesirable (including unhygienic) practices or techniques during the course of any of these activities and to assess the possible harmful effects by evaluation any cumulative, synergistic or potentiating effect of its use and the phenomenon of human intolerance to substances foreign to the body”.
- In the EU, current labeling regulations require packages with meat and meat products in MAP to be labeled with “Packaged in a protective atmosphere”. The specific gases do not need to be declared on the packages. Conventional gases will probably be the ones most concerned by this statement. Low concentrations of CO in food packaging systems could be required to appear on the product label to inform consumers of its use in the product.
- Analysis of CO can be used to control whether muscle food products have been treated with CO or not, despite not having been labeled as such. These considerations underline the suitability to develop better alternative fast methods to detect even small amounts of the CO-Mb adduct in muscle tissues, in regard to the fraudulent treatment of meat and fish in the MAP systems.

All this promotes high ethical standards in commercial communications by means of effective regulation, for the benefit of consumers and business in the world (Europe and beyond), and this implies that industrialized countries and members of their regulatory agencies must develop coherent and robust systems of regulation that can respond effectively to new challenges. 

## Figures and Tables

**Table 1:** Important findings in evaluating CO-modified atmosphere packaging (MAP) on extending the shelf-life of fresh meats.

Publication	Results/Conclusions
Luño et al. [88]	The presence of CO and 50% CO_2_ extends the shelf-life by inhibition of spoilage bacteria growth, delayed metmyoglobin (MetMb) formation; maintains red color and odor of fresh meat and slows down oxidative reactions. CO concentrations of 0.5–0.75% were able to extend shelf life of packaged fresh meat by 5–10 days at 1 °C.
Nissen et al. [89]	At 4 °C, the shelf-life of ground beef packed in MA, based on color stability and background flora development, was prolonged for the high CO_2_ (60%)/low CO (0.4%) mixture compared to high O_2_ packaging (70% O_2_/30% CO_2_), but at 10 °C (abuse temperature), the shelf-life was <8 days for both packaging methods. The growth of *Y*. *enterocolitica* and *L. monocytogenes* in ground beef stored in the high CO_2_/low CO mixture was not increased as a result of prolonging the shelf-life. However, the growth of strains of *Salmonella* at 10 °C in this mixture does emphasize the importance of temperature control during storage.
Sørheim et al. [85]	By adopting the use of CO in combination with high CO_2_ for meat packaging under MAP, retailers must adopt packaging systems indicating the deadlines for optimal use of the packaged product. However, as with other perishable foods in MAP, food products must be handled according to strict hygienic standards, and low storage temperatures must be maintained in a continuous chill chain.
Jayasingh et al. [90]	Ground beef packaged in 0.5% CO would maintain color stability for several weeks. The penetration of CO and depth of formation of COMb in the meat is dependent on the concentration of CO in the atmosphere, the time of CO exposure and the structure of the meat. For safety issue concerns, the workers were not exposed to dangerous levels of CO during MA packaging, which was verified by CO detectors.
Kusmider et al. [91]	The low levels of CO (<1%) incorporated into MAP will maintain a stable, cherry-red color along with extended shelf-life of irradiated ground beef during 28 days of storage, thus countering the potentially negative color effects of irradiation.
Krause et al. [92]	0.5% CO significantly improved color stability and sensory attributes for both injected and non-injected pork chops. The depth (bright red band: COMb) of CO penetration from the surface increases as exposure time increases. The depth of the COMb layer steadily increased from the surface to the interior of the chops during exposure to CO. 0.5% CO packages increased in penetration depth from 5 mm on Day 1 to about 10 mm at 14 days, 15 mm at 28 days and 25 mm at Day 36.
John et al. [93]	Raw ground beef packaged in 80% O_2_ maintained desirable bright red color until 10 days, but began to darken by Day 14 and lost all red color by Day 21. However, ground beef stored in highO_2_-MAP was very susceptible to premature browning (PB) during cooking. PB is a food safety concern, because the cooked product appears done at temperatures where food poisoning organisms may survive. Raw ground beef held in 0.4% CO remained bright red throughout the 21 days of storage. PB and rancidity associated with ground beef packaged in highO_2_-MAP were prevented by packaging in 0.4% CO.
John et al. [94]	Premature browning and rancidity associated with beef packaged in highO_2_-MAP were prevented by packaging in 0.4% CO, 30.3% CO_2_ and 69.3% N_2_.
Mancini et al. [13]	Packaging atmospheres containing high levels of O_2_ promote beef bone marrow discoloration. Exclusion of O_2_ from MA packages and the addition of low concentrations of CO (0.4%) minimized this discoloration by limiting hemoglobin oxidation through packaging atmosphere and will promote a bright red lumbar vertebrae color for as long as 6 weeks after packaging.
Martínez et al. [95]	The retention of color and odor of fresh pork sausages packaged in MA was better achieved using atmospheres containing low CO_2_ concentrations (20%). However, increasing concentrations of CO_2_ (60%) promoted Mb and lipid oxidation, despite the better antimicrobial effects promoted by the high level of CO_2_. The atmosphere containing 0.3% CO together with 30% CO_2_ maintained the red color for 20 days, but failed to keep the fresh odor longer than 16 days, in agreement with its small effect on Thiobarbituric Acid Reactive Substances (TBARS) formation and microbial growth.
Wilkinson et al. [84]	Use of CO in MAP provides sufficient shelf-life extension of at least 8 weeks of refrigerated retail-ready pork chops in a master-packaging system. The inclusion of CO in the master-packs has not inhibited the growth of pathogenic organisms. However, given the stable fresh color of CO-treated meat and the lack of inhibition of pathogen growth by CO, there is concern that CO-MAP under certain conditions may pose a food safety risk. As such, safe refrigeration and handling must be emphasized with this type of product.
Wicklund et al. [96]	Chops packaged in CO-MAP were redder and darker than chops packaged in HiO_2_-MAP. Based on sensory attributes, the CO-MAP pork was pinker than the HiO_2_ pork after cooking to an internal temperature of 70 °C. CO-MAP chops also experienced less purge loss than pork in HiO_2_-MAP, which may have contributed to the increased juiciness perceived by the panelists.
Sørheim et al. [97]	CO can be used as an alternative colorant to nitrite in meat products. A gas mixture containing 1% CO was sufficient for achieving a red/pink color of cooked or fermented meat products. Sausages with CO discolored faster during air and light display than nitrite controls. However, discoloration of CO sausages was reduced by anaerobic storage in darkness, showing that absence of O_2_ is a necessity for optimum color formation and stability of these sausages.
De Santos et al. [86]	Enhanced pork chops were packaged in 0.36% CO and stored at 4 °C for 0, 12, 19 or 26 days, displayed for 2 days, then cooked to six endpoint temperatures (54, 60, 63, 71, 77 and 82 °C). As storage time increased, Pork chops packaged in CO-MAP retained their internal pink color even after cooking to 82 °C.
Stetzer et al. [98]	Steaks were packaged in 0.4% CO/30% CO_2_/69.6% N_2_ or 80% O_2_/20% CO_2_, stored in the dark for 12 and 26 days and placed in a lighted retail display case. Steaks were visually evaluated by trained panelists. Steaks were cooked for consumer color evaluation. CO had no effect on flavor or acceptability and minimal effects on other characteristics, such as color, sheen and purge loss. If the CO environment provides microbiological stability through storage, it can be expected that the raw product appearance will not differ from steaks in traditional HiO_2_-MAP.
Aspé et al. [99]	Beef chops (longissimus dorsi) were pre-treated with 5% CO/24 h, vacuum packed and stored at 2 °C. Chops pre-treated with CO were redder during all of the storage period than controls without CO, and microbial shelf-life was 11 weeks. The pre-treatment did not affect pH, water-holding capacity, drip loss or rancidity of the meat stored in vacuum.
Mantilla et al. [100]	Color stability of tilapia fillets (*Oreochromis niloticus*) was significantly improved by pre-mortem CO treatment (CO-euthanized tilapia). The color of CO-treated fillets was also retained during frozen storage compared to untreated fillets. Hence, pre-mortem CO treatment could be used as a valuable method for improving the color of tilapia during storage.
Linares et al. [101,102]	The effect of the type of stunning (electrically vs. gas), MA and their interactions on meat quality of suckling lamb of the Spanish Manchego breed was determined at 7, 14 and 21 days of storage. Stunning by CO_2_ gas prevented the negative effects that electrical systems have on meat quality in lamb apparent during storage. Furthermore, a low CO (30% CO_2_/69.3% N_2_/0.7% CO) level could give the best meat quality characteristics, even at 3 weeks of storage in the electrically-stunned group. In addition, in the gas-stunned group, it is possible to obtain a product of better color and more tenderness with a post-packing life of 7 days and possibly 15 days using CO in the gas mixture.
Grobbel et al. [103]	Steaks packaged in HiO_2_ MAP discolored faster and to a greater extent than steaks packaged by vacuum package (VP) or ultra-low O_2_ with CO (ULO_2_CO) MAP. Non-enhanced muscles packaged by VP and ULO_2_CO MAP had more stable display color and very desirable tenderness and flavor compared with those packaged in HiO_2_ (80% O_2_/20% CO_2_).
Ramamoorthi et al. [104]	The combined irradiation with CO-MAP showed that, after 14 days of storage, aerobically-packaged beef was visually greener and less red than CO-MAP packaged beef. CO-MAP preserved color until 21 days of storage. CO-MAP could be also used to preserve color of beef irradiated at sufficient doses (~2 kGy) to reduce microbial loads to safe levels during 28 days of storage.
Mancini et al. [105]	Packaging steaks in CO (0.4% CO/30% CO_2_/69.6% N_2_) did not counteract the darkening effects of lactate enhancement. Nevertheless, CO improved color stability of beef steaks compared with high-oxygen packaging (80% O_2_/20% CO_2_).
Fontes et al. [106]	Fresh blood saturation with CO produces a dried blood of a pleasant pinkish-red color after 12 weeks of storage when packed in low O_2_ transmission rates (OTR) bags, with great potential as an additive in meat product formulations.
Raines and Hunt [107]	Increased CO concentration in combination with reduced headspace volume has a greater influence on COMb development. Smaller headspaces with higher concentrations of CO (i.e., 0.8% vs. 0.4% CO) optimize the package size while maintaining or improving the appearance of beef packaged in CO-MAP without compromising consumer safety. This would result in greater efficiency of case-ready meat distribution, making the CO-MAP system more economically feasible and advantageous.
Jeong and Claus [108]	The color of CO-packaged ground beef upon opening the package deteriorated with display time and became less red. However, the initial rate of color deterioration was faster in vacuum-packaged ground beef when it was opened compared to CO-MAP-packaged product. When a CO-packaged product is opened, this color deterioration would provide consumers with a visual indicator of freshness.
Bjørlykke et al. [109]	CO could increase animal welfare when used to slaughter salmon or other fish. Exposure of fish to CO also could improve the quality of products.
Suman et al. [110]	The incorporation of chitosan increased the interior redness of ground beef patties stored in CO-MAP (0.4% CO + 19.6% CO_2_ + 80% N_2_). This incorporation was also minimizes premature browning (PB) in patties stored under CO-MAP systems instead of under high-O_2_ MAP.
Ramamoorthi et al. [111]	Use of CO in MAP gasses has the potential to allow beef subjected to low doses of irradiation to retain its color.
Pivarnik et al. [112]	Filtered smoke (FS) presumably containing high % CO has been used to preserve taste, texture and/or color in tuna (*Thunnus albacares*). Therefore, a general statement indicating that FS treatments would extend shelf-life of tuna in the studied ways of storage: room temperature (21–22 °C), refrigerated (4–5 °C) and iced (0 °C).
Venturini et al. [113]	Packaging under 0.2% CO increased the color stability of beef steaks and ground beef for 28 days at 1 °C, even with residual O_2_ concentrations that are considered excessive for anaerobic packaging systems (above 0.1%). After 28 days of storage under CO-MAP and 24 h of air exposure, beefsteaks and ground beef maintained an acceptable appearance and a visual color similar or superior to that of fresh meat. However, after 24 h of air exposure, both the appearance and the smell of steaks and ground beef were considered “slightly unpleasant”.
Lavieri and Williams [114]	The CO-MAP (0.4% CO) treatment had no effect on maintaining the COMb “cherry red” fresh meat color during meat spoilage. No potential health hazards or deceptions were revealed due to simultaneous onset of spoilage and the presence of COMb “cherry red” fresh meat pigment in the CO-MAP. The CO absorbed in the meat ranged from 0.22–0.46 ppm CO/g of meat on Day 0 and increased to 2.08–2.40 ppm CO/g of meat on Day 25. The maximum level of CO detected in the meat in this study was below the Environmental Protection Agency (EPA) National Ambient Air Quality Standard of 9 ppm.
Liu et al. [115]	The CO-MAP (0.4% CO/30% CO_2_/69.6% N_2_) significantly increased red color stability of all muscles. Steaks in CO-MAP maintained a higher MetMb reducing activity (MRA) compared with those in HiO_2_-MAP during storage. After opening packages, the red color of steaks in CO-MAP deteriorated more slowly compared with that of steaks in HiO_2_-MAP.
Concollato et al. [116]	CO-treated fish resulted in an earlier onset of rigor mortis, lower final post-mortem muscle pH and higher drip loss after filleting. The assimilation of CO by Atlantic salmon’s muscles, through injection in the water, slightly increased lightness (L*) and yellowness (b*) values, limited however to the fresh samples. No significant difference in redness (a*) at any considered time was found between CO and the control group, probably because of the content of astaxanthin that may have minimized the color differences amongst the different groups.
Rogers et al. [117]	CO-MAP (0.4% CO, 30% CO_2_, 69.6% N_2_) exhibited more desirable color and consumer acceptability throughout lighted retail display of ground beef during 20 days.
Pereira et al. [118]	Addition of CO-treated blood allows the production of better-colored sausages (mortadella) having lower residual nitrite levels.
Fontes et al. [119]	Saturated porcine blood with CO (99%) could substitute meat by up to 20% for mortadella’s processing. Therefore, from the nutritional point of view, meat replacement with up to 20% of CO-treated blood is nutritionally adequate for being used in sausage production.
Yang et al. [120]	Aerobically-packaged beef steaks exhibited a bright-red color at the first 4 days. However, discoloration and oxidation became major factors limiting their shelf-life to 8 days. Compared with aerobic packaging, VP extended shelf-life of beef steaks, due to better color stability, together with lower oxidation and microbial populations. Among all packaging methods, CO-MAP (0.4% CO + 30% CO_2_ + 69.6% N_2_) had the best preservation for steaks, with more red color than other packaging types.
Sakowska et al. [121]	The raw steaks’ CO penetration depth increased as exposure times and CO concentration in gas mixtures increased. However, the COMb that formed did not always turn brown during thermal treatment. In cooked samples treated with 0.3% and 0.5% CO-MAP, a red COMb border was visible at the cross-section, whereas other CO packaging treatments had partial or total browning. To create a red color in raw beef and avoid a red boarder in cooked beef, up to 0.5% CO in VP and only 0.1% for MAP can be recommended.
Lyu et al. [122]	The pretreatment of CO combined with O_3_ at certain concentrations can be a promising technique to maintain the quality of beef meats under vacuum during storage.
Van Rooyen et al. [123]	The addition of CO pre-treatments prior to VP may be beneficial to allow a desirable color to be induced while allowing aging to occur within the package and increase meat tenderness. The 5-h CO pretreatment exposure time achieved the desirable color, and discoloration reached unacceptable levels by the use-by date. Therefore, applying 5% CO pretreatments may be a potential solution to current packaging issues within the meat sector for safety and enhancing meat quality. In addition, this anoxic packaging technology should prevent any negative quality issues related to high O_2_-MAP packaging.
Sakowska et al. [124]	Using CO significantly increased the brightness and the redness of beef steaks in both CO-vacuum packaging and CO-MAP systems during storage for 21 days. They evaluated the effects of 0.5% CO exposure in two MAP (0.5% CO + 30% CO_2_ + 69.5% N_2_), as compared with conventional VP, on the quality of packaged beef steaks stored for 21 days at 2 °C. The consumers have the greatest desire to purchase the vacuum-packed steaks after exposure in CO.
Van Rooyen et al. [125]	CO as a pretreatment applied prior to VP or VSP may play an important role in overcoming some of the challenges the meat industry faces. This technology provides a prolonged storage, improves the tenderness of the meat and prevents the negative problems associated with other packaging technologies (reduces the risk of the cross-linking/aggregation of myosin due to Hi-O_2_ MA, decreases energy usage, storage facilities and distribution costs).
